# Supplementary material for: A Critical View of the Application of the APEX Software (Aqueous Photochemistry of Environmentally-Occurring Xenobiotics) to Predict Photoreaction Kinetics in Surface Freshwaters
Source: Molecules. 2019 Dec 18;25(1):9. doi: 10.3390/molecules25010009 (PMC7017383; doi:10.3390/molecules25010009)
Supplement: Supplementary file 1 [file molecules-25-00009-s001.zip › 3.2.4_gcc-4.4.0.zip!/3.2.4_gcc-4.4.0/3.2.4_gcc-4.4.0/doc/octave/html/interpreter/Voronoi-Diagrams.html]

Voronoi Diagrams - Untitled


Next: Convex Hull,
Previous: Delaunay Triangulation,
Up: Geometry


---

### 29.2 Voronoi Diagrams

A Voronoi diagram or Voronoi tessellation of a set of points s in
an N-dimensional space, is the tessellation of the N-dimensional space
such that all points in v`(`p`)`, a partitions of the
tessellation where p is a member of s, are closer to p
than any other point in s. The Voronoi diagram is related to the
Delaunay triangulation of a set of points, in that the vertexes of the
Voronoi tessellation are the centers of the circum-circles of the
simplicies of the Delaunay tessellation.

— Function File: **voronoi** (x, y)  
— Function File: **voronoi** (x, y, "plotstyle")  
— Function File: **voronoi** (x, y, "plotstyle", options)  
— Function File: [vx, vy] = **voronoi** (...)  
> plots voronoi diagram of points `(`x`,` y`)`.
> The voronoi facets with points at infinity are not drawn.
> [vx, vy] = voronoi(...) returns the vertices instead of plotting the
> diagram. plot (vx, vy) shows the voronoi diagram.
>
> A fourth optional argument, which must be a string, contains extra options
> passed to the underlying qhull command. See the documentation for the
> Qhull library for details.
>
> ```
>             x = rand (10, 1);
>             y = rand (size (x));
>             h = convhull (x, y);
>             [vx, vy] = voronoi (x, y);
>             plot (vx, vy, "-b", x, y, "o", x(h), y(h), "-g")
>             legend ("", "points", "hull");
> ```
>
>
>
>
> **See also:** voronoin, delaunay, convhull.

— Function File: [C, F] = **voronoin** (pts)  
— Function File: [C, F] = **voronoin** (pts, options)  
> computes n- dimensional voronoi facets. The input matrix pts
> of size [n, dim] contains n points of dimension dim.
> C contains the points of the voronoi facets. The list F
> contains for each facet the indices of the voronoi points.
>
> A second optional argument, which must be a string, contains extra options
> passed to the underlying qhull command. See the documentation for the
> Qhull library for details.
>
>
>
> **See also:** voronoin, delaunay, convhull.

An example of the use of `voronoi` is

```
     rand("state",9);
     x = rand(10,1);
     y = rand(10,1);
     tri = delaunay (x, y);
     [vx, vy] = voronoi (x, y, tri);
     triplot (tri, x, y, "b");
     hold on;
     plot (vx, vy, "r");
```

Additional information about the size of the facets of a Voronoi
diagram, and which points of a set of points is in a polygon can be had
with the `polyarea` and `inpolygon` functions respectively.

— Function File: **polyarea** (x, y)  
— Function File: **polyarea** (x, y, dim)  
> Determines area of a polygon by triangle method. The variables
> x and y define the vertex pairs, and must therefore have
> the same shape. They can be either vectors or arrays. If they are
> arrays then the columns of x and y are treated separately
> and an area returned for each.
>
> If the optional dim argument is given, then `polyarea`
> works along this dimension of the arrays x and y.

An example of the use of `polyarea` might be

```
     rand ("state", 2);
     x = rand (10, 1);
     y = rand (10, 1);
     [c, f] = voronoin ([x, y]);
     af = zeros (size(f));
     for i = 1 : length (f)
       af(i) = polyarea (c (f {i, :}, 1), c (f {i, :}, 2));
     endfor
```

Facets of the Voronoi diagram with a vertex at infinity have infinity
area. A simplified version of `polyarea` for rectangles is
available with `rectint`


— Function File: area = **rectint** (a, b)  
> Compute the area of intersection of rectangles in a and
> rectangles in b. Rectangles are defined as [x y width height]
> where x and y are the minimum values of the two orthogonal
> dimensions.
>
> If a or b are matrices, then the output, area, is a
> matrix where the i-th row corresponds to the i-th row of a and the j-th
> column corresponds to the j-th row of b.
>
>
>
> **See also:** polyarea.

— Function File: [in, on] = **inpolygon** (x, y, xv, xy)  
> For a polygon defined by `(`xv`,` yv`)` points, determine
> if the points `(`x`,` y`)` are inside or outside the polygon.
> The variables x, y, must have the same dimension. The optional
> output on gives the points that are on the polygon.

An example of the use of `inpolygon` might be

```
     randn ("state", 2);
     x = randn (100, 1);
     y = randn (100, 1);
     vx = cos (pi * [-1 : 0.1: 1]);
     vy = sin (pi * [-1 : 0.1 : 1]);
     in = inpolygon (x, y, vx, vy);
     plot(vx, vy, x(in), y(in), "r+", x(!in), y(!in), "bo");
     axis ([-2, 2, -2, 2]);
```
